# Nanofabricated high turn-density spiral coils for on-chip electromagneto-optical conversion

**DOI:** 10.1038/s41378-024-00674-9

**Published:** 2024-03-25

**Authors:** Ilhan Bok, Alireza Ashtiani, Yash Gokhale, Jack Phillips, Tianxiang Zhu, Aviad Hai

**Affiliations:** 1 Department of Biomedical Engineering, University of Wisconsin–Madison, Madison, WI USA; 2 Department of Electrical and Computer Engineering, University of Wisconsin–Madison, Madison, WI USA; 3 Wisconsin Institute for Translational Neuroengineering (WITNe), University of Wisconsin–Madison, Madison, WI USA

**Keywords:** Sensors, Nanoscale devices

## Abstract

Circuit-integrated electromagnets are fundamental building blocks for on-chip signal transduction, modulation, and tunability, with specific applications in environmental and biomedical micromagnetometry. A primary challenge for improving performance is pushing quality limitations while minimizing size and fabrication complexity and retaining spatial capabilities. Recent efforts have exploited highly involved three-dimensional synthesis, advanced insulation, and exotic material compositions. Here, we present a rapid nanofabrication process that employs electron beam dose control for high-turn-density diamond-embedded flat spiral coils; these coils achieve efficient on-chip electromagnetic-to-optical signal conversion. Our fabrication process relies on fast 12.3 s direct writing on standard poly(methyl methacrylate) as a basis for the metal lift-off process. Prototypes with 70 micrometer overall diameters and 49–470 nm interturn spacings with corresponding inductances of 12.3–12.8 nH are developed. We utilize optical micromagnetometry to demonstrate that magnetic field generation at the center of the structure effectively correlates with finite element modeling predictions. Further designs based on our process can be integrated with photolithography to broadly enable optical magnetic sensing and spin-based computation.

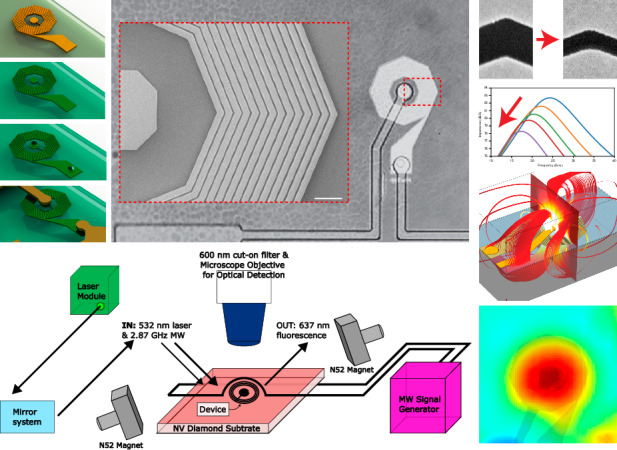

## Introduction

**Fig. 1:**
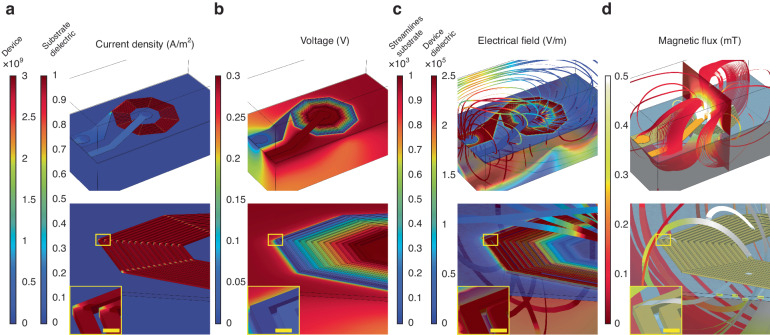
Modeled electromagnetic behavior of the device. Nanofabricated spiral coil design and predicted performance on high resistivity substrate are evaluated via finite element analysis in response to the input current injected through the dielectric and the coil center. **a** Stimulation-driven current density (A/m^2^) in the device is predictably greater than that in the surrounding substrate and dielectric material. Bottom and inset, enlarged regions demonstrating the details of individual coil turns. **b** Corresponding voltage (V) reaches 0.3 V in response to the current density shown in (**a**). **c** Resulting electrical field (V/m) in the substrate and device dielectric. **d** Magnetic flux (mT) is determined when the current injection reaches > 0.5 mT in the coil center. Streamlines are tangent to the vector field representing the electrical field within the model space. Shown are cross-sections of the model space and streamlines around the device. Scale bars are 1 µm for all insets in (**a**)-(**d**). Interturn spacings (*s*) ranging between 49 and 470 nm are demonstrated in the remainder of this study

On-chip micro- and nanofabricated inductors, antennas, and electromagnets extend the possibility of compartmentalizing a wide variety of technologies and research applications^1–3^. From wireless communication^4–6^, high-frequency signal conversion, power transfer and filtering^7–11^, to environmental and biological sensing^2,12–15^, new designs are leveraging diverse geometries and material compositions to transform electromagnetic energy over a broad spatiotemporal range. Specific lab-on-chip platforms for magnetic detection and manipulation rely on patterned coils and loops for optical magnetometry^16,17^, nuclear magnetic resonance (NMR) spectroscopy and imaging^18,19^, magnetic particle separation^20–23^, molecular magnetophoresis^24–26^, and cell manipulation and labeling^27–30^. Theoretical limitations and fabrication constraints restrict performance and are closely related to quality factor, frequency bandwidth, and temporal response^31–34^. More recent innovative devices have demonstrated improved properties by utilizing approaches such as three-dimensional fabrication;^35–38^ mechanically self-assembled coils^38–41^; air-core or air-suspended coils^35,42–44^; and alternative materials such as graphene, carbon, ZnO and others^45–49^. However, complex designs are not easily integrated into standard fabrication processes. Consequently, metal-based flat spiral coils remain the mainstay devices for on-chip electromagnetic signal conversion owing to the relatively small number of lithography steps and higher structural and thermal stability. Spiral inductors have been integrated into numerous recent applications, including power harvesting components on flexible and bioresorbable electronic sensors^50–54^, recording and stimulation devices for wireless neurological applications^14,55–59^ and ingestible electroceuticals^60,61^. Additionally, they empower modalities such as nuclear magnetic resonance (NMR) and biomedical magnetic resonance imaging (MRI) by providing high spatial resolution microprobes for spectroscopy and imaging^18,19^. While most systems employ microlithography to pattern coil structures, a small number of studies have begun exploring nanoscale lithography to increase the spatial features while maximizing performance. These include electron beam lithography (EBL) for synthesizing meandering inductors with submicron conducting lines^62^, complementary metal-oxide semiconductor (CMOS)-compatible glancing angle physical vapor deposition (GLAD) for vertically aligned nanohelices^63^, and spiral patterns achieved via focused ion beam fabrication (FIB)^64^. The emerging integration of these methods and other promising nanofabrication techniques^65–67^ with standard CMOS processes, particularly EBL, highlights opportunities for designing novel rapid fabrication processes for high spatial resolution electromagnetic conversion. In this study, we introduce a high-density nanofabricated spiral coil design for on-chip electromagnetic signal conversion (Fig. 1). Modeling predictions of this design allow predictions of current density (Fig. 1a), surface potential (Fig. 1b), electric field (Fig. 1c) and magnetic flux density (Fig. 1d) for a given input, and information on the proper nanofabrication parameters for optimized performance. Using a simplified EBL fabrication process that relies on dose control to achieve high turn-density nanocoils, our process utilizes proximity effect in poly(methyl methacrylate) (PMMA) exposure and metal lift-off to demonstrate tightly packed coils with minimal PMMA collapse; these coils achieve 49.7 nm turn spacing and an inductance of up to 12.8 nH for 10 turn prototypes. Using diamond-based optical magnetometry in conjunction with finite element computational modeling, we demonstrate efficient magnetic field generation at the center of the structure. Further designs based on this process can be integrated with photolithography to broadly strengthen electromagnetic circuits for magnetic sensing and modulation.

## Results

**Fig. 2:**
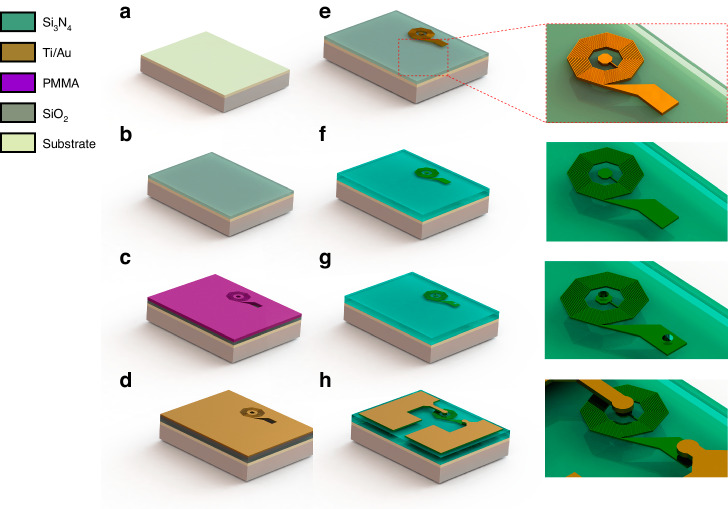
Fabrication process overview. **a** The device is fabricated on resistive glass or diamond substrates. **b** A 100 nm thick SiO_2_ barrier layer is deposited on the surface. **c** A 400 nm PMMA layer is spin coated and patterned via EBL to define the nanocoil features. **d** A Ti/Au (6/60 nm) bilayer is deposited via e-beam evaporation and subsequently lifted off to create a coil structure (**e**). **f** An additional insulating SiO_2_ layer is deposited to prepare for PL micropatterning via electrode routing. **g** Via holes are defined via PL and etched with a fluorine-based plasma recipe. **h** Finally, patterns for electrode traces are fabricated, and gold electrodes are laid via evaporation and lift-off. The samples are further packaged and routed onto glass printed circuit boards for magnetometry

**Fig. 3:**
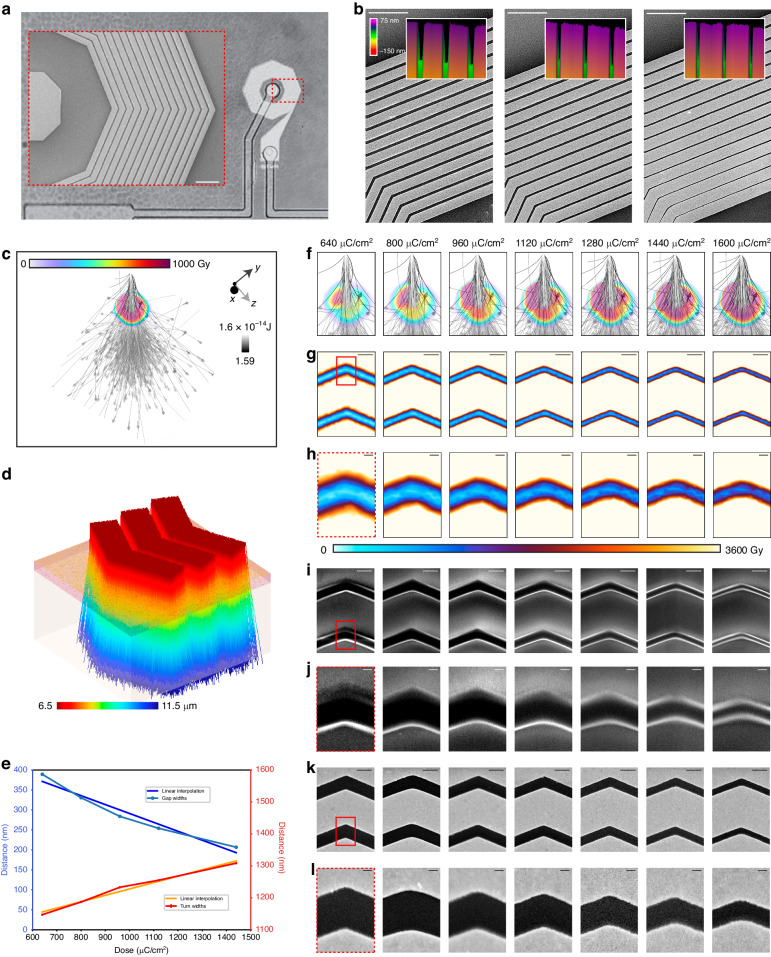
Dose-dependent feature analysis of nanofabricated spiral coils. **a** A complete view of the end device. The dotted red region is a scanning electron microscopy (SEM) image of the EBL step in the left inset, showing distinct uniformity of turns for a dose of 1120 µC/cm^2^ (scale bar = 5 µm). Different doses with the closest corresponding AFM traces are shown in (**b**): 960 µC/cm^2^ (left), 1280 µC/cm^2^ (center), and 1600 µC/cm^2^ (right; scale bar = 5 µm), with corresponding 3-dimensional AFM traces color-coded by height (insets). **c** Single-pixel Monte Carlo simulation of PMMA exposure where 1 Gy ≡ 7.366 eV/cm^3^ and **d** corresponding triple-turn simulation color-coded by particle trajectory height. The gold, magenta, and cream-colored layers correspond to gold, PMMA, and quartz, respectively. **e** Average turn widths and gap widths versus dose, determined using atomic force microscopy, showing a linear relationship between dose and feature size. Shown on the right is a matrix comparing (**f**) single-pixel Monte Carlos, **g** multiturn Monte Carlos, **h** (with zoomed regions of interest), **i** PMMA pre-liftoff, **j** (with close-up ROIs), and **k** Ti/Au post-liftoff, **l** (with close-up ROIs). The columns from left to right correspond to doses of 320, 480, 640, 800, 960, 1120, 1280, 1440, and 1600 µC/cm^2^ (scale bar = 500 nm (overview panels) or 100 nm (zoomed ROIs))

Following fabrication process development (Fig. 2), we synthesized spiral nanocoils using a broad electron beam dose matrix ranging between 320 and 1600 µC/cm^2^ (Fig. 3). The end device (Fig. 3a) exhibited a minimum turn spacing (*s*) of 197.8 nm for a dose of 1120 µC/cm^2^ (Fig. 3a, inset). We utilized the proximity effect of an identical pattern to control the nanocoil turn width (*w*) and *s* (Fig. 3b) while maintaining a constant turn density. As shown in Fig. 3b on the left, middle and right subpanels, doses of 800, 1440 and 1600 µC/cm^2^ yielded minimum *s* values of 329.6, 300.3 and 139.2 nm, respectively; and an average Ti/Au slope of 50.98 ± 1.14 degrees (1.23 ± 0.05 nm_(z)_/nm_(x)_) was determined from the AFM analysis (Fig. 3b, insets). To predict the relationship between dose and *s*, we used finite element modeling of electron trajectories in PMMA (Fig. 3c, d) demonstrating good correlation (R = 0.96887) between simulated dose response (Fig. 3f–h) and s in PMMA following development (Fig. 3i, j). The mean predicted area for a single exposed dot at 2 nA and 100 keV for a dose of 320 µC/cm^2^ (Fig. 3f, leftmost panel) was 6.38 nm^2^ and increased linearly to 8.71 nm^2^ for a dose of 1600 µC/cm^2^ (Fig. 3f, rightmost panel). The resulting average *s* following lift-off ranged between 206.7 and 389.2 nm (Fig. 3k, l) for doses ranging between 1440 and 640 µC/cm^2^. For this range, an inverse linear relationship was observed between dose and *s* (Fig. 3e) corresponding to a surface resistance ranging between 349.58 and 397.16 kΩ/m for the same metal film thickness across all devices.

**Fig. 4:**
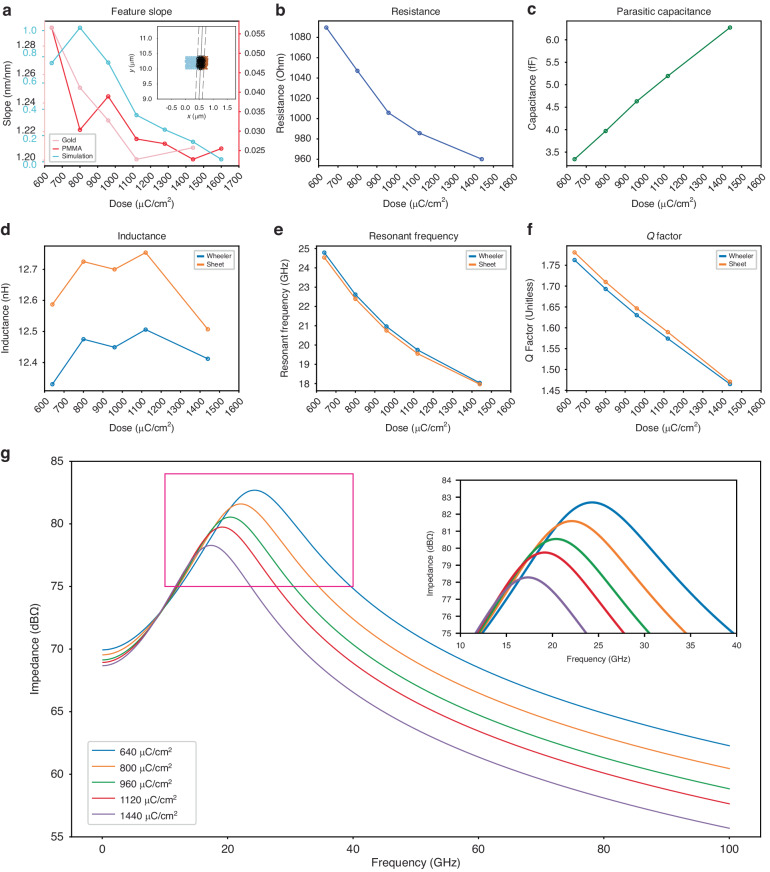
Nanocoil feature measurements and fitness calculations. **a** Finite element analysis (cyan) reveals slope trends in agreement with the AFM (pink) and SEM (red) measurements. The inset shows an example of support vector machine (SVM) regression of a PMMA slope. Blue and orange dots represent mesh points with subthreshold and suprathreshold exposure, respectively. The solid line and dashed lines represent the optimal hyperplane and margin, respectively. **b** Whole-coil resistance and **c** parasitic interturn capacitance. Wheeler method and sheet spiral inductance calculations are shown in (**d**), with the corresponding self-resonance (**e**) and Q factor (**f**). All quantities are plotted versus the electron beam dose ranging from 640 to 1440 µC/cm^2^ except for the SEM data, which extend to 1600 µC/cm^2^. Comparing the impedance in decibel ohms to the linear frequency reveals a dose-dependent tuning curve (**g**) with varying resonant frequency and Q factor (inset)

A high current amplitude range during normal device operation can result in high ohmic loss and a localized hot region with changes as small as 10 mK at an ambient temperature equivalent to a magnetic field change of ∼30 nT, resulting in reduced accuracy^68^. Dose selection can be used to mitigate these alterations in device characteristics by adapting impedance and turn density to the expected current range and maximizing coil fill factor^69^. This can be achieved by increasing the dose of electron beam exposure (Fig. 4). Whole-coil resistance (Fig. 4b), parasitic interturn capacitance (Fig. 4c), Wheeler^70^ and sheet^69^ spiral coil inductance estimates (Fig. 4d), and corresponding Q factor (Fig. 4e) and self-resonance (Fig. 4f) data are plotted with respect to the dose. Q factor and resonance frequency can be tuned by dose selection, with lower doses suitable for improved Q factor, but the resistive heating is increased. For the resonance frequencies shown here (Fig. 4g), impedance was the major contributor to higher loss, whereby Q was reduced by 16.9%, from 1.76 (1.78) to 1.46 (1.47) according to Wheeler (Sheet) estimates for doses of 1440 µC/cm^2^ and 640 µC/cm^2^ and corresponding self-capacitance of 3.3 fF and 6.3 fF and resistance of 1089.8 Ω and 960.1 Ω, respectively. In addition to performance considerations, dose control presents possibilities for frequency tuning (Fig. 4e, g) demonstrating average frequency shifts of 0.92 ± 0.36 GHz per 100 µC/cm^2^.

A relatively constant inductance is concomitant with evenly spaced coil turns and serves as a foundation for optimizing all other parameters. Measurements of turn slope using atomic force microscopy (Fig. 4a, pink) and scanning electron microscopy (Fig. 4a, red) showed that the slope asymptotically decreased with increasing dose, in agreement with our Monte Carlo finite element analysis model. Vertical slanting has the potential to impact heat dissipation and capacitive coupling, enabling further optimization of coil behavior for in vivo applications. A dose-dependent tuning curve was generated by comparing the impedance in decibel ohms to the linear frequency (Fig. 4g) with varying resonant frequency and Q factor (Fig. 4g, inset).

**Fig. 5:**
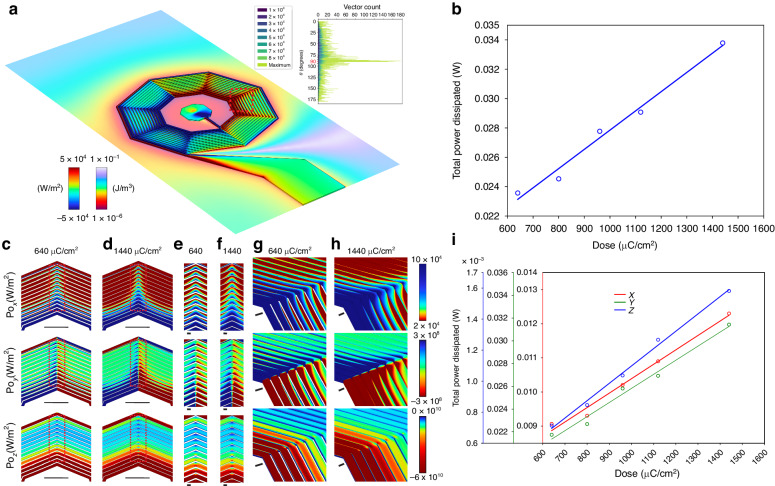
Dose-dependent power dissipation quantification and analysis. **a** Full view of the nanocoil (dose: 640 µC/cm^2^) with the coil surface color coded by the x-component of the Poynting vector (Po_x_) and the plane of the coil color coded by the magnetic energy density (J/m^3^). The red dashed box shows the regions expanded in (g) and (h). Inset: Subtraction histogram between high-dose (1440 µC/cm^2^) and low-dose (640 µC/cm^2^) vector counts versus angle color-coded by magnitude. **b** Vector magnitude for the integral of the X, Y, and Z components ${||}\mathop{\oint\oint}\nolimits_{{coil}}\,{{Po}}_{x}\,\hat{x}+\mathop{\oint\oint}\nolimits_{{coil}}\,{{Po}}_{y}\,\hat{y}+\,\mathop{\oint\oint}\nolimits_{{coil}}\,{{Po}}_{z}\,\hat{z}{||}$ over the entire coil surface of dissipated power versus dose examined with lines of best fit affirming a linear trend between the electron beam dose-dependent turn width and dissipated power. **c**–**h** Two-dimensional (2D) Poynting vector x (Po_x_), y (Po_y_) and z (Po_z_) plots for 640 µC/cm^2^ (**c**, **e**) and 1440 µC/cm^2^ (**d**, **f**) for the dashed boxes in (**a**) (**c**, **d** scale bar = 10 µm) and zoomed inset (red dashed boxes, **e**, **f**, scale bar = 1 µm). Three-dimensional (3D) plots of corresponding regions for 640 µC/cm^2^ (**g** scale bar = 1 µm) and 1440 µC/cm^2^ (**h** scale bar = 1 µm). **i** Separate surface integrals of the X, Y, and Z components of the Poynting vector demonstrating linear relationships between the electron beam dose-dependent turn width and dissipated power

To fully explore the parameter space of coil characteristics, we quantified dose-dependent power dissipation using our finite element analysis (Fig. 5). A full view of the nanocoil (Fig. 5a) corresponding to a dose of 640 µC/cm^2^ with the coil surface color coded by the x-component of the Poynting vector (Po_x_) and the plane of the coil color coded by magnetic energy density (J/m^3^) shows significant stratification within the coil and gradients outside the coil for a 3 mA applied current. The red dashed box in Fig. 5a corresponds to regions expanded in Fig. 5g, h. A subtraction histogram between the high-dose (1440 µC/cm^2^) and low-dose (640 µC/cm^2^) vector counts versus angle color-coded by magnitude, revealed stark differences in directionality surrounding 90 degrees corresponding to the plane of the coil (Fig. 5a, inset). Integrating the vector magnitudes of the X, Y, and Z components ${||}\mathop{\oint\oint}\nolimits_{{coil}}\,{{Po}}_{x}\,\hat{x}+\mathop{\oint\oint }\nolimits_{{coil}}\,{{Po}}_{y}\,\hat{y}+\,\mathop{\oint\oint}\nolimits_{{coil}}\,{{Po}}_{z}\,\hat{z}{||}$ of dissipated power versus dose over the entire coil surface demonstrated a linear trend between the electron beam dose-dependent turn width and dissipated power (Fig. 5b). Two-dimensional (2D) Poynting vector x (Po_x_), y (Po_y_) and z (Po_z_) plots for 640 µC/cm^2^ (Fig. 5c, e, g) and 1440 µC/cm^2^ (Fig. 5d, f, h) showed significant changes in power distribution with dose. Separate surface integrals of the X, Y, and Z components of the Poynting vector also confirmed linear relationships between the electron beam dose-dependent turn width and dissipated power (Fig. 5i). These results provide a basis for optimizing the power characteristics of nanofabricated coils on diamonds.

**Fig. 6:**
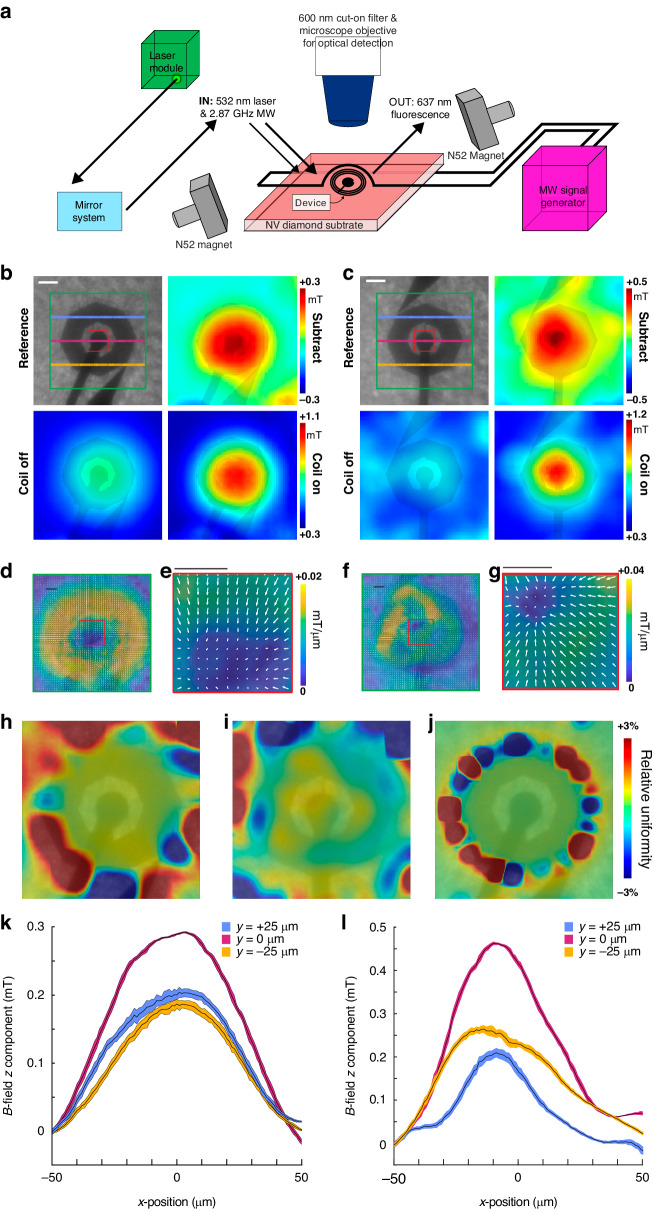
Optical magnetometry measurements of the nanocoil B-field strength during current injection. **a** Experimental setup. **b**, **c** B-field maps of two different device routing configurations. Top left: reference image with delineated line scans; bottom left: B-field amplitude (0 mA DC); bottom right: B-field amplitude (3 mA DC); top right: current OFF minus current ON subtraction; B-field z component. **d**–**g** Vector field lines of the B-field gradient overlaid on B-field maps. **e** and **g** orrespond to dashed boxes in (**d**) and (**f**), respectively. **h**, **i** Uniformity maps comprising vector field lines of B-field gradient overlaid on corresponding B-field gradient maps. **j** Simulation of uniformity map for comparison. **k**, **l** 100 µm line scans of the B-field z component for (**b**) and (**c**), respectively, taken at *y* = +25, 0, −25 µm from the device center. Scale bars = 20 µm

To quantify the B-field strength developing in the device in response to injected current and to verify conformity with our modeling predictions, we used optically detected magnetic resonance (ODMR) micromagnetometry to form an electromagnetic-to-optical junction on-chip (Fig. 6). Nanocoils embedded in nitrogen vacancy (NV) diamond samples were excited at 532 nm and RF-irradiated at frequencies ranging between 2.78 and 2.96 GHz in the presence of a DC magnetic field (0.8 mT) (Fig. 6a). We compared two different routing configurations to account for the electrode path contributions to the magnetic field (Fig. 6b, c, upper left panels). RF spectra were acquired along with optical collection at 637 nm with and without current injection (bottom left and bottom right panels in Fig. 6b, c, respectively). The mean amplitudes of the magnetic B-field strength across the nanocoil were 0.18 ± 0.08 mT (Fig. 6b) and 0.28 ± 0.003 mT (Fig. 6c) for the two configurations, demonstrating no significant difference (p = 7.06·10^−5^ across an 80 µm diameter field of view surrounding the coil center). Respective B-field maps predicted by finite element simulations showed a mean B-field amplitude of 0.08 ± 0.03 mT; this result effectively agreed with the experimental measurements (R = 1.2·10^−2^). To confirm sufficient magnetic field uniformity across the coil surface, we quantified the magnetic field gradients (Fig. 6d–g) to generate corresponding uniformity maps from ODMR measurements (Fig. 6h, i) and compared our measurements to finite element simulation results (Fig. 6j). We found that the uniformity within the sensing region of the coil was within ±1.2%, with regions outside the coil experiencing slightly greater field inhomogeneity (<±3%); these results were consistent with our simulation results. An improved NV layer surface tolerance is predicted to yield higher uniformity below ±1%. Finally, we measured a maximum B-field of 0.25 ± 0.03 mT (Fig. 6k) and 0.38 ± 0.08 mT (Fig. 6l) at the center of the nanocoils for both configurations, occupying an area of 1.26·10^3^ µm^2^ and corresponding to a predicted maximal B-field of 0.47 ± 0.13 mT interpolated over a 1 µm pixel size in the model.

## Discussion

Based on these results, the electromagnetic-to-optical junction mediated by nanofabricated spiral coils can facilitate new possibilities for diamond-based optical magnetometry^16,17^ and emerging optical NMR detection schemes^71,72^. Using our protocol for careful control of self-resonance in conjunction with the integration of additional capacitive elements, future standalone devices can be specifically tuned to 2.87 GHz frequencies for efficient (on resonance) in situ RF irradiation of the NV diamond layer-embedded nanocoils. Furthermore, the utilization of dedicated pulse sequences with modified photonic and RF temporal signatures can be used to increase contrast and sensitivity^73,74^. Combined with tuned resonance, this type of configuration is expected to increase RF energy harvesting and greatly improve ODMR sensitivity, with the potential to broadly impact the quantum information storage and computing by incorporating substrate-integrated patterned nanocoil arrays to augment increase spin-based computational elements.

## Materials and methods

### Nanofabrication process development

The fabrication process consisted of three main steps: (1) high-fill-factor nanocoil electron beam lithography (EBL) on insulating substrates; (2) photolithography (PL) for micropatterning of nanocoil contacts and die routing; and (3) wire bonding and encapsulation onto a glass printed circuit board for micromagnetometry measurements. We used a standard lift-off process (Fig. 2) on either glass (76.22 mm Borofloat 33, 500 µm thick wafers) or diamond (3.6 × 3.6 mm electronic grade 400 µm thick samples, 10 µm ~3.8 ppm nitrogen vacancy layer, Element six, Santa Clara, CA). The samples were cleaned using isopropyl alcohol (IPA) and dried with N_2_ (100%) for EBL preparation (Fig. 2a) followed by deposition of a 100 nm SiO_2_ barrier layer (Fig. 2b) by plasma-enhanced chemical vapor deposition (PECVD) (PlasmaTherm 73/72, Saint Petersburg, FL) at a chamber temperature of 250 °C, 810 square cubic centimeters per minute (SCCM) N_2_O, 440 SSCM 2% silane, 900 mT pressure and 36 W RF power at a 100 s deposition time. During process development on the diamond samples, the barrier layer was etched and redeposited several times using buffered oxide etch (BOE 6:1) for 10 minutes and rinsed with deionized (DI) water, with no observed injury to nitrogen vacancy (NV) diamond layer or bulk substrate. Following deposition, samples were spin coated (Fig. 2c) with PMMA 950 K A7 (M230002, Kayaku Advanced Materials Inc., Westborough MA) at 3600 rotations per minute (RPM) for 60 s and then baked at 180 °C for 2 min to yield a typical resist thickness of 400 nm. We used EBL (Elionix ELS G-100, 100 keV, 2 nA, 2.5 nm pixel size, 0.01–0.05 µs dwell time for doses 320-1440 µC/cm^2^, 500 µm field size) to test a high beam exposure matrix for overexposing nanocoil features to increase turn width and narrow turn spacing (*s*) in the resist during development. The samples were developed with IPA:MIBK (2:1) (2 min). A dose of 800 µC/cm^2^ reproducibly yielded 330 nm spacing. Based on EBL optimization and subsequent PMMA development, the samples were rinsed with IPA and DI water and dried with N_2_ prior to metal deposition and lifted off (Fig. 2d, e, respectively). After a 10 s oxygen plasma treatment of the descum surface (YES R3 Plasma Asher, 250 W, 80 SCCM O_2_), a Ti/Au (6/60 nm) metal bilayer was deposited via e-beam evaporation without breaking vacuum (Fig. 2d). The nanocoil was then lifted off in an ultrasonic bath at a medium-high vibration rate with remover (Microposit Remover 1165, Kayaku Advanced Materials, Inc., Westborough, MA) for 10 minutes (Fig. 2e) followed by rinsing with DI water and N_2_ drying. An additional 400 nm SiO_2_ insulating layer (Fig. 2f) was deposited via PECVD (250 °C, 810 SCCM N_2_O, 440 SSCM 2% silane, 900 mT pressure, 36 W RF, 100 s deposition time) as a pre-step for micropatterning of contacts by PL.

### Contact micropatterning and outside routing

Since nanofabrication is the only dimensionally critical step in our process, our protocol involves the use of optical microlithography for fabricating electrode contacts to connect the coil to outside circuitry. The insulated substrate was spin coated with S183 photoresist (30 s, 3000 RPM, 1.3 µm thickness) baked at 110 °C for 1 min, followed by soft contact lithography (Karl Suss MA6, 9.5 s exposure time, 10 mW/cm^2^ broadband mercury lamp) to pattern via holes at the coil interface pads (Fig. 2g). Samples were developed in MF-321 (Kayaku Advanced Materials, Inc., Westborough MA) for 60 sec, washed with DI water and dried with N_2_. Holes were etched in an RIE chamber with a CF4-based recipe (PlasmaTherm 790, chamber temperature 40 °C, 45 SCCM CF_4_, 5 SCCM O_2_, 40 mT pressure, 100 W RF power, etch time of 360 m), which was subsequently washed with DI water and dried with N_2_ to expose the nanocoil metal contact pads (Fig. 2g, inset). This was followed by an additional lithography step and lift-off process to generate electrode traces for on-chip routing (Fig. 2h) using APOL-LO 3204 negative photoresist (KemLab, Inc., Woburn, MA, USA) spin-coated at 4000 RPM for 30 s and soft baked at 110 °C for 1 min. Contact PL was performed with a 15 s exposure time (Karl Suss MA6, 10 mW/cm^2^) followed by postexposure at 110 °C for 1 min. Finally, the sample was developed with MF321 for 2 min, rinsed with DI water and dried with N_2_. To verify that via walls were covered with gold and to create reliable contact with the nanocoil contact pads, a 400 nm gold layer was evaporated using e-beam, and lift-off was performed with the recipe used for the PMMA/Ti/Au lift-off process described above for nanocoil fabrication. Printed glass circuit boards for die wire bonding were fabricated on a 3 × 2-inch glass substrate (Fisherbrand™ Extra-Thick Microscope Slides, 1.2 mm thick) using APOL-LO 3204 photoresist with identical recipes used for contact micropatterning, as described above: A Ti/Au (10/200 nm) layer was deposited using e-beam evaporation followed by a lift-off procedure. To bond the die to the glass printed circuit board (PCB), we used SU-8 2002 (Kayaku Advanced Materials, Inc., Westborough, MA) by depositing a small (1 µl) droplet on the glass surface at the chip bonding area, followed by hot plate curing at 95 °C for 5 min to homogenize the SU-8 temperature. The die was then placed on the PCB on the hotplate on top of the non-crosslinked SU-8 for 5 min to let viscous SU-8 gradually reflow under the die. Subsequently, to create a permanent bond, the sample was subjected to UV flood exposure and a postexposure bake at 95 °C for 5 min. Next, a manual gold wire bonder (KS-4524, K&S, Fort Washington, PA) was used to connect the electrodes to the PCB gold traces. Finally, gold traces on the PCB were connected either directly or with coaxial SubMiniature version A (SMA) adapters (#132134-10; Amphenol Connex, Wallingford, CT) with conductive silver ink cured at room temperature for 15 min.

### Optical micromagnetometry

Optically detected magnetic resonance (ODMR) micromagnetometry was used to visualize the magnetic field in devices utilizing a 50 mW 532 nm laser (OBIS 532-80 LS 1264453, Coherent, Santa Clara, CA) in NV diamond-embedded nanocoils. A printed microwave antenna (1 mm diameter) was used to deliver RF to the NV layer underneath the coils in conjunction with laser excitation. Microwave signals were generated using an RF signal generator (SG 384, Stanford Research Systems, Sunnyvale, CA) fed through an RF amplifier (Mini-circuits ZHL-16W-43-S+, Scientific Components Corp., Brooklyn, NY) connected to the antenna. A direct current (DC) bias magnetic field was applied using a 1.48 T magnet (B333-N52, KJ Magnets, Pipersville, PA) placed approximately 4 inches away from the sample. The bias field at the sample was estimated to be 0.8 mT. Fluorescence signal changes during current injection at the device were captured using an upright microscope (SM-LUX HL, Leica Biosystems, Wetzlar, Germany) mounted with a CMOS camera (CS165MU1, Thorlabs, Inc., Newton, NJ) operating at 12 frames/sec and a resolution of 720 × 540 pixels with a corresponding region of interest (ROI) size of 527 × 395 µm. A total of 181 frames surrounding resonance at ~2.87 GHz were acquired while sweeping between 2.78 and 2.96 GHz at 1 MHz intervals for a total of 181 data points per pixel and an acquisition time of 15 min. The image capture and delivery of microwaves and lasers were directly controlled through a MATLAB (MathWorks, Inc. Natick, MA, USA) interface and in-house routines. The magnetic field strength was converted from microwave stimulation frequency as follows:

1 $$\Delta E={g}_{L}{\mu }_{B}{m}_{j}B$$

2 $$B=\frac{2\pi h\nu }{g{\mu }_{B}}$$

where *ΔE* is the magnetic interaction energy due to the Zeeman effect, *μ*_*B*_ is the Bohr magneton, *m*_*j*_ is the total angular momentum, and *B* is the magnetic field. The native noise level was quantified across all sweeps per measurement.

### Atomic force microscopy

A Bruker Dimension Icon Atomic Force Microscopy (AFM) system operating in tapping mode with TESPA-V2 tips was used to scan the nanocoils. A 50 µm wide field of view (FOV) was surveyed at a scan rate of 0.1 Hz to reduce tip and sample wear and improve image quality. The scans were imported into Bruker Nanoscope Analysis 2.0, sectioned, and exported as XZ plane height maps for further processing using Python. Turn width and spacing were determined using a partition threshold of 40 nm above the previous gap minimum. Mean turn width and spacing for all 14 turns and 13 gaps were plotted for comparison.

### Finite element analysis

Simulations of electron beam dose trajectories and device magnetic response were performed in COMSOL multiphysics simulation environment (COMSOL, Inc., Stockholm, Sweden). Optimized coil pattern described previously^59^ and used here for nanofabrication was imported to COMSOL and extruded 500 nm along the z-axis. The dielectric layer was 900 nm thick above the substrate, providing 400 nm of separation between the device and the electrode contacts. Cylinders with a diameter of 8 µm were extruded through the dielectric layer over the interface pad and ground pad, and microfabricated electrode contacts were patterned above the dielectric layer and extruded to a thickness of 400 nm. The device had 14 turns, resulting in an open core percentage of 46.2%. The electrical properties of the device and microfabricated electrode contacts were set to those of gold: ε = 1, μ = 1, and σ = 45.6 ·10^6^ S/m. The electrical properties of the substrate and dielectric layer in the model were set to those of silicon dioxide (SiO_2_): ε = 4.2, μ = 1, and σ = 1e-15 S/m. The electrical properties of the space above the device were set to those of air: ε = 1, μ = 1, and σ = 0.7 S/m. All device geometries were tested using an input current of 3 mA. The current was applied through the outside face of the microfabricated electrode contact connecting to the interface pad at the center of the inductor. The outside face of the electrode contact above the ground pad was used as the ground port.

Monte Carlo modeling of the electron trajectory and sample exposure was performed using the charged particle tracing (cpt) module. A pattern consisting of three 1.02 μm wide nanocoil turns was constructed within a simulation arena defined as a 7 × 7 × 7 μm^3^ region of vacuum. A base layer of quartz glass 3 μm thick with ε = 4.2, σ = 1e-14 S/m, and ρ = 2210 kg/m^3^ was used as the substrate. A layer of 400 nm thick Microchem 950 PMMA resist was placed on top of the substrate with σ = 1.10^−19^ S/m and ρ = 1180 kg/m^3^. A titanium surface with ε = 1·10^100^ (virtually ∞) and σ = 2.6e6 S/m was assigned to the top of the PMMA. We applied an inlet boundary condition with 3056 randomly positioned particles per release with 1000 releases over a period of 32 ms. The particles had an initial kinetic energy of 100 keV. A normally distributed random velocity component normalized to 5% of the Z component velocity was applied in the X and Y directions. The electron interactions within PMMA were modeled using particle matter interactions with a cutoff energy of 8.6 eV, with both nuclear stopping and ionization loss sub nodes. A cutoff screening angle of 0.1 degrees and an electronic stopping power of 4 (MeV cm^2^)/g were used for each condition. We also applied velocity reinitialization to the PMMA, with a 40% likelihood of one secondary particle having an equal speed as the primary particle but a randomly chosen direction being released. The interaction of particulate matter in the quartz layer was modeled using nuclear stopping at a cutoff angle of 0.1 degrees. We applied an electric current (ec) module with the substrate initially grounded to accurately portray current discharge by grounding the bottom of the substrate. Within this module, a 10 nm thick layer of conductive electrical shielding was used to model titanium.
